# Safety and Effectiveness of Volumetric Modulated Arc Therapy-Based Stereotactic Radiosurgery for Posterior Fossa Brain Metastases: A Single-Centre Experience

**DOI:** 10.3390/jcm14238540

**Published:** 2025-12-02

**Authors:** José Manuel Sánchez-Villalobos, Alfredo Serna-Berna, Juan Salinas-Ramos, Pedro Pablo Escolar-Pérez, Ginés Luengo-Gil, Marina Andreu-Gálvez, Emma Martínez-Alonso, Miguel Alcaraz

**Affiliations:** 1 Department of Neurology, University Hospital Complex of Cartagena, 30202 Cartagena, Murcia, Spain; 2 Department of Cell Biology and Histology, School of Medicine, Regional Campus of International Excellence, Campus Mare Nostrum, IMIB-Pascual Parrilla, University of Murcia, 30100 Murcia, Spain; emma@um.es; 3 Department of Medical Physics and Radiation Protection, University Hospital Complex of Cartagena, 30202 Cartagena, Murcia, Spain; alfredo.serna@carm.es; 4 Department of Radiation Oncology, University Hospital Complex of Cartagena, 30202 Cartagena, Murcia, Spain; juan.salinas@carm.es (J.S.-R.); pedrop.escolar@carm.es (P.P.E.-P.); 5 Health Sciences Faculty, Universidad Católica de Murcia (UCAM), 30107 Guadalupe, Murcia, Spain; 6 Group of Molecular Pathology and Pharmacogenetics, Pathology Department, IMIB-Pascual Parrilla, University Hospital Complex of Cartagena, 30202 Cartagena, Spain; 7 Department of Otorhinolaryngology, Head and Neck Surgery, Reina Sofia University Hospital, 30003 Murcia, Spain; marina.andreu@um.es; 8 Department of Radiology and Physical Medicine, School of Medicine, Regional Campus of International Excellence, Campus Mare Nostrum, IMIB-Pascual Parrilla, University of Murcia, 30100 Murcia, Spain; mab@um.es

**Keywords:** posterior fossa metastases, VMAT, stereotactic radiosurgery, complications, local control, brainstem, procedure time, neuro-oncology

## Abstract

**Background/Objectives:** Posterior fossa brain metastases (PFBMs) pose particular risks owing to their proximity to the brainstem and fourth ventricle. We evaluated the safety (treatment-related complications), local effectiveness, and procedural efficiency of volumetric modulated arc therapy (VMAT)-based stereotactic radiosurgery (VMAT-SRS) for PFBMs. **Methods**: This single-centre, retrospective study derived a PFBM subgroup from an overall institutional cohort of 123 patients treated with VMAT-RapidArc SRS/fSRS. The doses were 12–20 Gy (single fraction) or 5 × 6 Gy (selected cases). Local response (mRECIST) and predefined safety endpoints (symptomatic oedema with brainstem/IV-ventricle compromise, obstructive hydrocephalus, haemorrhagic transformation, CSF diversion, and urgent neurosurgery) were assessed. Overall survival and procedural time were analysed. **Results**: Thirty-one patients (39 lesions) were included; 76.9% of them received single-fraction SRS. In addition, 74.2% of patients had supratentorial metastases with posterior fossa involvement. Kaplan–Meier overall survival at 6, 12, 24, and 48 months was 74%, 58%, 26%, and 9.7%, respectively; the median survival time was 12.6 months. Among evaluable lesions, local control was 84.5% (per-lesion response: 15.5% PD, 28.1% SD, 34.4% PR, and 22.0% CR). No clinically significant posterior fossa local complications were observed. Three patients developed radiation-induced leukoencephalopathy after whole-brain radiotherapy (WBRT) and radiosurgery for synchronous supratentorial metastases. The median procedural time was 25.0 min (IQR 9.0) with one isocentre versus 52.5 min (IQR 9.75) with two. **Conclusions:** VMAT-SRS/fSRS for PFBMs achieved high local control, very low posterior fossa toxicity, and favourable procedural efficiency, supporting its use as a safe, rapid, frameless alternative to WBRT and other radiosurgical platforms such as Gamma Knife in appropriately selected patients.

## 1. Introduction

Brain metastases (BMs) are the most common intracranial tumours, affecting 20–40% of cancer patients, and their incidence has increased owing to advances in imaging and systemic therapies [1,2,3]. Traditionally, whole-brain radiotherapy (WBRT) has been the standard treatment; however, its use has been limited by the risk of neurocognitive decline. In contrast, stereotactic radiosurgery (SRS) provides survival outcomes comparable to those of WBRT while significantly reducing neurocognitive toxicity [4,5]. Conceptually, WBRT is primarily used for global intracranial disease control, delivering a relatively low-to-moderate biologically effective dose (BED) per lesion, whereas focal techniques such as SRS deliver ablative high BED to discrete metastases, with better sparing of the normal brain (e.g., WBRT 30 Gy/10 fractions, BED_10_ = 39 Gy; SRS 20 Gy/1 fraction, BED_10_ = 60 Gy) [6,7,8]. Among the available platforms, volumetric modulated arc therapy (VMAT) has been rapidly adopted owing to its efficiency, frameless delivery, and shorter procedure times, making it an increasingly accessible technique within radiation oncology departments [9].

In our previous VMAT-SRS cohort, approximately 16% of brain metastases involved the posterior fossa (cerebellum/brainstem), with breast cancer predominating in this compartment [9]. This infratentorial location is clinically high-risk because of the limited cranial reserve and proximity to the brainstem and fourth-ventricle outflow, predisposing patients to acute obstructive hydrocephalus and sudden neurological deterioration [10]. The infratentorial compartment is a critical region for space-occupying lesions; even small volumetric increases can precipitate brainstem compression or fourth-ventricle obstruction. While stereotactic radiosurgery is effective in this setting, it carries location-specific hazards, including brainstem injury (dose/volume-related), treatment-related oedema or haemorrhagic transformation with mass effect on critical structures, and occasional need for rescue neurosurgical procedures such as external ventricular drainage or ventriculoperitoneal shunting.

Modern series focused on cerebellar, brainstem, and fourth-ventricle metastases report low but non-negligible rates of obstructive hydrocephalus and CSF diversion after SRS, alongside isolated cases of acute hydrocephalus from peritumoural oedema and rare post-SRS haemorrhage [11]. However, most of these studies involve radiosurgical or radiotherapeutic management with a Gamma Knife, and evidence regarding linear accelerator (LINAC)-based VMAT approaches remains limited [11,12,13,14,15,16,17]. In this context, the aim of our study was to evaluate the safety—defined by treatment-related complications—and effectiveness—measured in terms of local response and procedural efficiency—of posterior fossa metastases treated with VMAT RapidArc stereotactic radiosurgery [13]. Demonstrating a favourable safety profile for VMAT-SRS would add pragmatic advantages, including shorter procedural times and frameless delivery, which can improve patient comfort and streamline the clinical workflow. Moreover, the broad availability of LINAC may facilitate its wider adoption and resource-efficient implementation in routine practice.

## 2. Materials and Methods

### 2.1. Study Design

A cross-sectional observational study was performed through a retrospective review of patients with posterior fossa brain metastases (PFBMs) treated with VMAT-based stereotactic radiosurgery (VMAT-RS) between October 2012 and January 2018 [9]. The cohort was further expanded by including patients with breast cancer (BC) and PFBMs from a complementary institutional series up to July 2018 [18], resulting in a total of 123 patients treated with VMAT-based radiosurgery. All patients with at least one PFBM were selected, yielding a final study cohort of 31 patients. Treatments were performed at the Department of Radiation Oncology of the University Hospital Complex of Cartagena (Spain). The data collection and observational study was approved by the Ethics Committee of the University Hospital Complex of Cartagena (Spain).

### 2.2. Study Cohort

The following variables were collected: sex, age, primary tumour, presence of extracranial metastatic disease, total number of brain metastases (BMs) at the time of radiosurgery, local treatments before and/or after SRS, and Karnofsky Performance Status (KPS). BMs were classified according to their location in the different structures of the posterior fossa of the cerebellum and brainstem (midbrain, pons, and medulla oblongata). Early post-treatment complications following radiotherapy were collected for each BMs and classified as follows: (1) symptomatic haemorrhagic transformation (defined as causing increased peritumoural oedema, compressive effects on brainstem structures or the fourth ventricle (IV ventricle), or acute neurological symptoms; (2) local radiotoxicity presenting as new-onset or markedly increased peritumoural oedema; (3) secondary obstructive hydrocephalus. Beyond early events, late radiological complications attributable to SRS delivered for PFBMs or prior radiotherapy (for example, WBRT) were systematically collected with a minimum follow-up of 12 months. Survival time was measured from the date of the patient’s first radiosurgical treatment for PFBMs.

The gross tumour volume (GTV), planning target volume (PTV), prescribed dose, and treatment regimen (single fraction or hypofractionated) were also collected. The assessment of local control to treatment was performed by applying the modified Response Evaluation Criteria in Solid Tumours (mRECIST) 1.1 [19]. The local response was analysed individually for each BM being classified into one of the following categories: progressive disease (PD), stable disease (SD), partial response (PR), and complete response (CR). The mRECIST 1.1 criteria were used for local response assessment of each BM [19]. Neuroimaging follow-up was performed at a mean of 2.8 months (SD 1.6) and was performed with MRI. Imaging was not available in 17.9% of cases.

### 2.3. Treatment Technique

The treatment protocol was based on our previously reported methodology [9]. GTVs were contoured on gadolinium-enhanced 3D T1-weighted MRI, with a 2 mm margin added to define the PTV. Patients were immobilised using a frameless thermoplastic mask, and all treatments were delivered with VMAT using RapidArc technology (Varian Medical System, Palo Alto, CA, USA), consisting of five non-coplanar arcs of 6 MV photons on a Varian iX linear accelerator with a multileaf collimator. Plans were optimised with Eclipse v10.0 (AAA algorithm, 1.2 mm grid) to achieve ≥99% PTV and 100% GTV coverage in SRS, or ≥98% in fSRS. Cone Beam CT (CBCT) was performed for image-guidance positioning.

### 2.4. Statistical Analysis

Statistical analyses were performed using IBM SPSS Statistics for macOS, version 25.0 (IBM Corp., Armonk, NY, USA). Continuous variables were summarised as mean ± SD or median (IQR), and groups (SRS vs. fSRS) were compared using Welch’s *t*-test for unequal variances. Categorical variables were compared using Pearson’s χ^2^ or Fisher’s exact test when expected counts were <5. Time-to-event outcomes were estimated using Kaplan–Meier, reporting medians and 95% CIs; pointwise survival at 6, 12, 24, and 48 months was also calculated. Univariable Cox proportional hazards models were fitted for the selected covariates (for example, KPS, primary tumour, extracranial metastases), with hazard ratios (HRs) and 95% CIs reported. All tests were two-sided with α = 0.05.

## 3. Results

### 3.1. Cohort Characteristics

Thirty-one patients with PFBMs comprising thirty-nine individual lesions were selected for the present analysis. The median age at treatment was 58 years (range, 38–82 years), and the cohort consisted of 17 males (55%) and 14 females (45%). The most frequent primary tumours were lung cancer (total *n* = 19, non-small cell lung carcinoma, *n* = 18; small cell lung carcinoma, *n* = 1), breast cancer (*n* = 10), and others (*n* = 2). At the time of radiosurgical treatment, 77.4% of the patients had a KPS of 70 or higher. The lesion distribution was as follows: cerebellum (*n* = 35; 89.7%) and brainstem (*n* = 4, 10.3%; all of them in the Varolio’s pons). Of the lesions, 76.9% were treated with single-fraction SRS, whereas 23.1% received a fractionated regimen (fSRS). The median GTV was 0.7 cm^3^ (IQR 2) and PTV was 2.5 cm^3^ (IQR 4.2). GTV and PTV were significantly lower in patients treated with SRS than in those treated with fSRS (Welch’s *t*-test: GTV, *p* = 0.041; PTV, *p* = 0.030). All patients underwent VMAT–RapidArc stereotactic radiosurgery. For single-fraction SRS, the prescribed doses ranged from 12 to 20 Gy; fractionated SRS (5 × 6 Gy) was employed in patients with prior WBRT, lesions adjacent to critical structures, or larger target volumes. Extracranial metastases were present in 67.7% of patients with posterior fossa brain metastases versus 55.4% of those without; the difference was not statistically significant (Pearson’s χ^2^ = 1.447, *p* = 0.229). In addition, at least two patients developed leptomeningeal carcinomatosis during the follow-up, and one patient harboured two satellite lesions adjacent to a primary cerebellar metastasis. The main characteristics of the patients and BMs treated are summarised in Table 1.

### 3.2. Local Control and Overall Survival

Among the evaluable lesions, the local control rate was 84.5%. The per-lesion response rates were 15.5% for PD, 28.1% for SD, 34.4% for PR, and 22.0% CR. The median procedural time was 25.0 min (IQR 9.0; 95% CI 21.83–28.28) with a single isocentre, compared with 52.5 min (IQR 9.75; 95% CI 46.69–58.31) with two isocentres.

**Figure 1:**
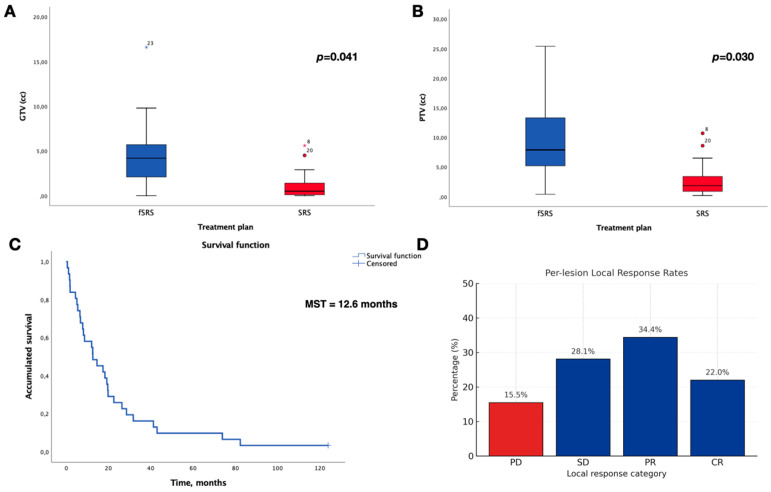
Tumour volume treated, overall survival, and local responses. (**A**) GTV; (**B**) PTV. Boxplots show the treated tumour volumes for fSRS (blue) and SRS (red). Circles represent outliers and asterisks represent extreme values as defined by SPSS; the colour of the symbols corresponds to the treatment group (blue for fSRS, red for SRS). (**C**) Overall survival of the cohort of patients with PFBMs. (**D**) Summary of local response per mRECIST criteria: disease progression (PD, red) and disease control (SD/PR/CR, blue).

**Figure 2:**
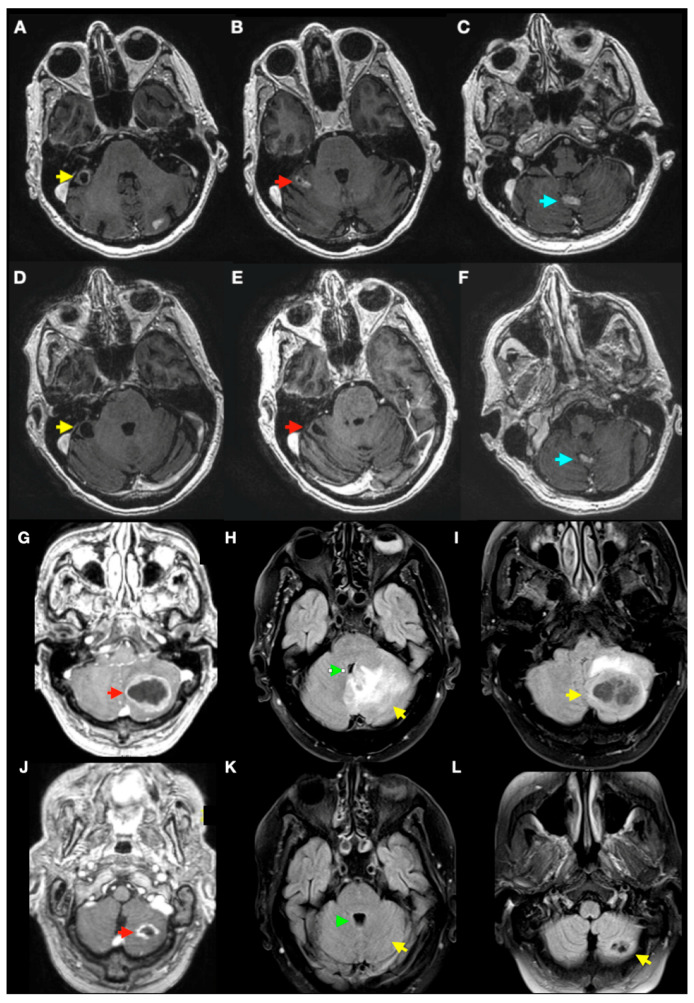
Cerebellar BMs MRI images before and after VMAT-SRS treatment. Axial 3D T1-weighted post-gadolinium images of posterior fossa brain metastases (PFBMs) before and after VMAT-SRS. For each case, the top row shows pre-treatment and the bottom row post-treatment. Case 1 (**A**–**F**): patient with multiple posterior fossa metastases. ((**A**,**D**), yellow arrows): flocculonodular lesion with cystic change and enhancing mural/irregular components pre-SRS (**A**); on follow-up (**D**), contrast enhancement has resolved, leaving a malacic cavity. ((**B**,**E**), red arrows): right cerebellar hemispheric metastasis with irregular enhancement pre-SRS (**B**); on control imaging (**E**), the enhancement has disappeared with a small residual malacic cavity. ((**C**,**F**), blue arrows): vermian metastasis pre-SRS (**C**) showing a partial response on follow-up (**F**) with reduction in size and enhancement. Case 2 (**G**–**L**): patient with a very large left cerebellar hemispheric metastasis causing marked fourth-ventricle collapse pre-SRS (**G**–**I**); post-SRS images ((**J**–**L**), two months later): significant tumour size reduction (red arrowheads; images (**G**,**J**)), marked reduction in oedema (yellow arrowheads; images (**H**–**L**)), and resolution of fourth-ventricle collapse (green arrowheads; images (**H**,**K**)).

**Figure 3:**
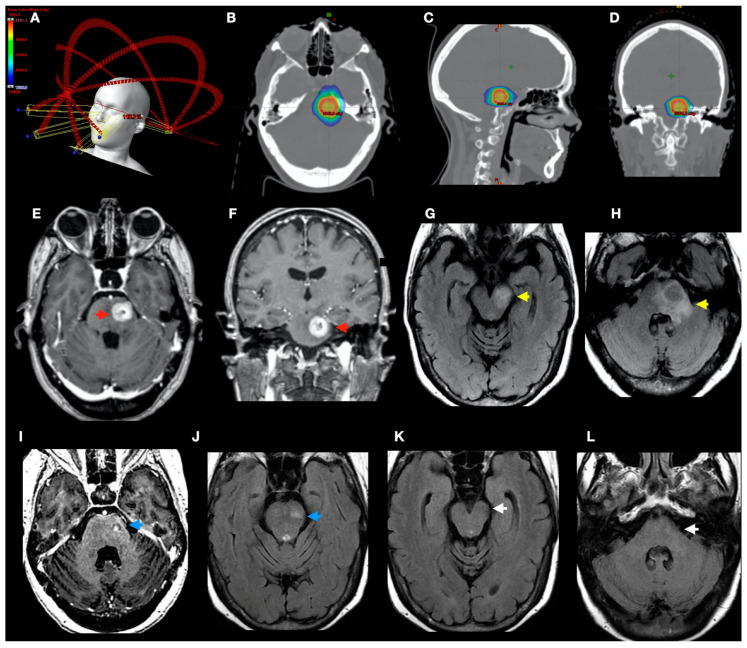
Treatment planning with VMAT-fSRS and local response. (**A**) Planning example of a patient with one brain metastasis in the pons, treated with fSRS 5 × 6 Gy. Images (**B**–**D**) represent axial, sagittal, and coronal plane rotations, respectively. Red PTV outline, green 25 Gy isodose, light blue 20 Gy isodose and dark blue 15 Gy. Pre-treatment MRI (**E**–**H**): On post-contrast T1WI, a well-circumscribed lesion (1.7 × 1.7 cm) in the left hemipons shows peripheral ring enhancement consistent with a necrotic metastasis (red arrows, (**E**,**F**)). FLAIR images show extensive vasogenic oedema extending along the adjacent cerebellar peduncle and into the ipsilateral pons and midbrain (yellow arrows, (**G**,**H**)). Post-treatment MRI (3 months later, (**I**–**L**)): On post-contrast T1WI, the left hemipontine lesion decreased significantly with heterogeneous nodular enhancement, and it remained hyperintense on T2/FLAIR (blue arrows, (**I**,**J**), respectively). The vasogenic/perilesional oedema resolved across all previously involved regions (white arrows, (**K**,**L**)).

Based on the Kaplan–Meier analysis of patients with PFBMs, the median survival time (MST) was 12.6 months (SE = 4.86; 95% CI, 3.07–22.13). The overall survival rates at 6, 12, 24, and 48 months after treatment were 74%, 58%, 26%, and 9.7%, respectively. In the univariable Cox regression analysis, KPS > 70 was a significant predictor of overall survival (hazard ratio [HR] = 0.14; 95% confidence interval [CI], 0.05–0.40; *p* < 0.001), indicating a substantially lower hazard among patients with higher KPS. Neither the primary tumour type (*p* = 0.282) nor the presence of extracranial metastases (*p* = 0.130) was associated with overall survival. Figure 1, Figure 2 and Figure 3.

### 3.3. Radiological Complications of SRS Treatment

**Figure 4:**
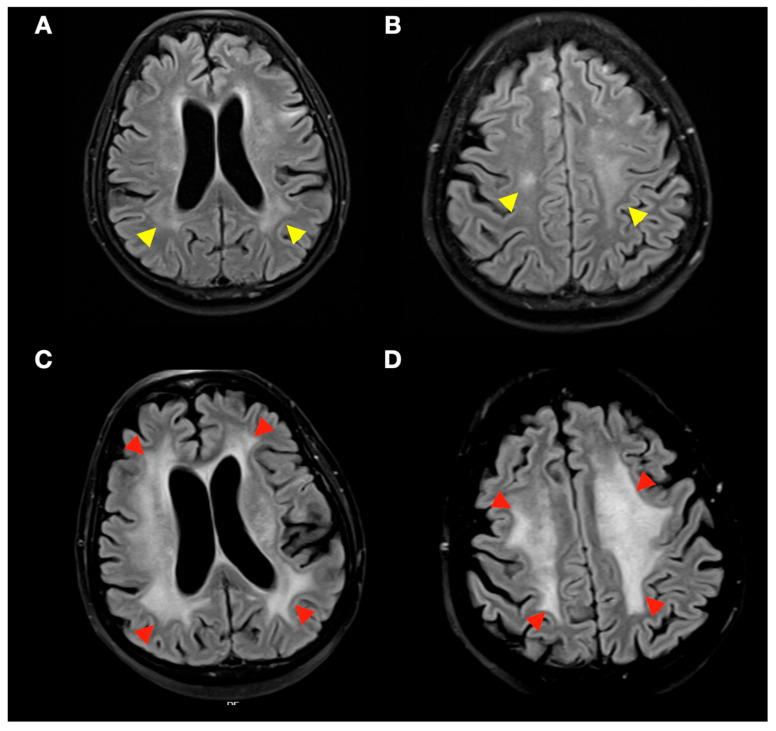
Treatment planning with VMAT-fSRS and treatment response. Axial FLAIR MRI. (**A**,**B**) (**top**): Yellow arrowheads indicate incipient signs of radiation-induced leukoencephalopathy after prior WBRT. (**C**,**D**) (**bottom**): Red arrowheads show progression after SRS in patients with synchronous infratentorial and supratentorial treatment. Predominant involvement of the white matter is seen in the corona radiata (**A**,**C**) and centrum semiovale (**B**,**D**).

No clinically significant treatment-related complications were observed. There were no cases of significant increase in peritumoural oedema with fourth-ventricle compression, obstructive hydrocephalus, or symptomatic haemorrhagic transformation of posterior fossa lesions, and no patient required CSF diversion (VP shunt/EVD) or urgent neurosurgical intervention. The only adverse events observed were three cases of radiation-induced leukoencephalopathy (Figure 4) in patients with prior WBRT, presenting as progression of supratentorial white-matter changes after radiosurgery for synchronous supratentorial metastases. A fourth patient developed probable radiation leukoencephalopathy attributable to prior WBRT. Importantly, the posterior fossa targets analysed in this study showed no clinically significant early local toxicity.

## 4. Discussion

In this single-centre series, we observed no clinically significant acute local complications following VMAT-based SRS for PFBMs. In the infratentorial compartment, where small volumetric changes can precipitate brainstem compression or fourth-ventricle obstruction, modern radiosurgical reports describe low but non-negligible rates of obstructive hydrocephalus, occasional CSF diversion, and rare post-SRS haemorrhage, findings drawn largely from Gamma Knife cohorts focused on cerebellar/brainstem/fourth-ventricle disease [12,13,15,16,20,21,22,23]. Therefore, our results provide LINAC-VMAT-specific safety data that complement the existing literature. Simultaneously, advances in systemic therapy (targeted agents, immune checkpoint inhibitors) are extending survival for patients with brain metastases, increasing cumulative neurotoxicity risk, and underscoring the value of organ-sparing local strategies that preserve cognition and function in these patients. Although hippocampal-sparing WBRT mitigates cognitive decline, focal SRS remains preferable when feasible. Above all, because we now know that hippocampal structures are not the only ones involved in memory or other cognitive functions [5,24]. Within this contemporary context, the frameless, widely available LINAC VMAT workflow—and its efficiency gains with single-isocentre non-coplanar delivery—adds practical relevance without compromising conformality or target coverage [25,26]. Regarding fractionation, single-fraction SRS was used to treat three of four lesions (76.9%), whereas hypofractionated SRS was used for larger volumes, with significant differences between groups (GTV *p* = 0.041; PTV *p* = 0.030). This pattern reflects volume-driven fractionation in the posterior fossa while maintaining an overall favourable safety profile.

Local control outcomes were favourable as a secondary endpoint. For the evaluation of the local response of BMs to radiosurgery treatment, the mRECIST criteria [19,27] were applied, representing an institutional adaptation of the RECIST 1.1 criteria [28]. One of the main differences between the two criteria is the definition of measurable lesions [19]. Per-lesion local control was 84.5%, which is consistent with contemporary Gamma Knife radiosurgery (GKRS) series in the posterior fossa/brainstem, typically reporting early local control rates of around 90%, and is also comparable to the outcomes of our previously published institutional VMAT-based SRS series (2012–2018, *n* = 229 BMs), in which early local control was 88.5% [9]. In our cohort, median overall survival was 12.6 months, with OS at 6, 12, 24, and 48 months of 74%, 58%, 26%, and 9.7%, respectively, figures that align with contemporary posterior fossa/brainstem radiosurgery series reporting median OS of 6–16 months, depending on case mix, dose/fractionation, and brainstem involvement [29,30,31].

We observed radiation-induced leukoencephalopathy in a small subset of patients, all with prior WBRT and radiosurgery for synchronous supratentorial metastases; one additional probable case was attributed to prior WBRT. This pattern reinforces two points: (i) the posterior fossa targets treated with VMAT-SRS in our cohort did not generate clinically or radiologically significant local toxicity; (ii) the whole-brain dose from prior WBRT remains a key driver of late leukoencephalopathy, supporting judicious WBRT use and, when necessary, hippocampal-avoidance strategies. This is particularly relevant in our cohort, where 74.3% of patients harboured synchronous supratentorial metastases, which often necessitates serial focal treatments and thereby increases cumulative neurotoxicity risk.

Looking ahead, HyperArc VMAT (HA-VMAT) and related single-isocentre multiple-target (SIMT) automation are likely to further reduce procedure times and standardise plan quality, enabling efficient treatment of many targets in a single session without strict numeric limits, provided that geometric eligibility and dosimetric constraints are met. Early clinical–technical series demonstrate feasible accuracy, rapid delivery, and promising plan quality for multiple lesions, supporting the role of HA-VMAT as a next-generation solution for complex intracranial disease distributions, including the posterior fossa [25,32]. At our institution, HA-VMAT has already been implemented, and preliminary experience indicates streamlined workflows and shorter in-room/procedural times, reinforcing its practical value for posterior fossa cases within a single-isocentre multitarget framework.

We acknowledge the following study limitations: this retrospective, single-centre design and modest sample size constrain multivariable analyses and widen confidence intervals for overall survival and local control; additionally, a small proportion of lesions lacked evaluable imaging follow-up data. Nonetheless, the primary endpoint was safety, for which a robust signal was detected. Future studies should be prospective and multicentre, harmonise brainstem dose–volume constraints, and ideally include head-to-head comparisons with GKRS—particularly for fourth ventricle and vermian location—while also evaluating procedural efficiency after the adoption of HA-VMAT radiosurgery. In addition, potential synergistic toxicities with systemic therapies should be assessed, for example, particularly with immune checkpoint inhibitors, which are relatively novel agents with neurotoxic potential and an increasingly widespread use in routine practice.

As a future direction, our group is currently extending this series in a prospective longitudinal study after HA-VMAT implementation to assess overall survival, local control, workflow efficiency and the impact of current systemic therapies. Therefore, this study serves as a starting point for subsequent comparative analyses and will also be expanded to primary posterior fossa tumours.

## 5. Conclusions

VMAT-SRS achieved high local control, very low infratentorial toxicity, and favourable procedural efficiency in PFBMs, supporting its use as a safe, rapid, frameless strategy in this critically sensitive region of the CNS, comparable to GKRS and consistent with the performance observed for VMAT–RapidArc in supratentorial disease.

## Figures and Tables

**Table 1:** Baseline clinical and treatment characteristics.

**Age, Years**	Median (IQR)	58	(12)
**Sex, *n* (%)**	Female	14	(45.2)
	Male	17	(54.8)
**Primary Tumour, *n* (%)**	Total cohort	31	(100)
	Non-small cell lung carcinoma	27	(58)
	Small cell lung carcinoma	1	(3.2)
	Breast	10	(32.2)
	Remainder	2	(6.6)
**KPS Score, *n* (%)**	70–100	24	(77.4)
	<70	7	(22.6)
**Overall Survival, Months**	Median (CI 95%)	12.6	(3–22.1)
**Extracranial Metastases, *n* (%)**	Yes	21	(67.7)
	No	10	(32.3)
**Radiosurgery Treatment, *n* (%)**	Total BMs treated	39	(100)
	SRS	30	(76.9)
	Fractionated SRS (fSRS)	9	(23.1)
Previous Treatment, *n* (%) *	None	20	(64.5)
	WBRT	10	(32.3)
	Surgery	1	(3.2)
Posterior Treatment, *n* (%) *	None	18	(58.1)
	WBRT	6	(19.4)
	Single or Fractionated SRS	10	(32.3)
	Surgery	0	(0.0)
**Patients with PFBMs ±** **Supratentorial BMs,** ***n*** **(%)**	Only PFBMs Treated	8	(25.8)
	PFBMs + Supratentorial BMs	23	(74.2)
**Gross Tumour Volume, cc** **(GTV)**	SRS, Median (IQR)	0.5	(1.3)
	fSRS, Median (IQR)	4.2	(6.6)
**Planning Target Volume, cc** **(PTV)**	SRS, Median (IQR)	1.9	(2.7)
	fSRS, Median (IQR)	7.9	(13.7)

* Individual patients could receive more than one treatment modality before and/or after radiosurgery.

## Data Availability

The datasets generated and/or analysed during the present study are not publicly available due to privacy and ethical restrictions related to patient confidentiality. Further information may be available from the corresponding author upon reasonable request.

## Abbreviations

The following abbreviations are used in this manuscript:

BED	Biologically effective dose
BM(s)	Brain Metastasis(es)
CBCT	Cone-Beam Computed Tomography
CI	Confidence Interval
CR	Complete Response
CSF	Cerebrospinal Fluid
EVD	External Ventricular Drainage
fSRS	Fractionated Stereotactic Radiosurgery
GKRS	Gamma Knife Radiosurgery
GTV	Gross Tumour Volume
HA (HyperArc)	Varian HyperArc VMAT platform
HR	Hazard Ratio
IQR	Interquartile Range
KPS	Karnofsky Performance Status
LINAC	Linear Accelerator
MRI	Magnetic Resonance Imaging
OS	Overall Survival
PD	Progressive Disease
PFBM(s)	Posterior Fossa Brain Metastasis(es)
PR	Partial Response
PTV	Planning Target Volume
RA (RapidArc)	Varian RapidArc VMAT delivery
SD	Stable Disease
SE	Standard Error
SRS	Stereotactic Radiosurgery
VMAT	Volumetric Modulated Arc Therapy
WBRT	Whole-Brain Radiotherapy
